# Commercialization Report: QUEL Imaging

**DOI:** 10.1117/1.BIOS.3.4.040501

**Published:** 2026-11-11

**Authors:** Ethan LaRochelle, Alberto Ruiz

**Affiliations:** QUEL Imaging, White River Junction, Vermont, United States

## Abstract

This community report discusses the experience of navigating the complex path of commercializing biomedical technologies, offering lessons for the scientific community and industry stakeholders alike.

## 1 Company Overview and Founding Team

We founded QUEL Imaging in 2019 while completing our PhDs at Dartmouth’s Thayer School of Engineering. The company grew out of Brian Pogue’s laboratory, where we were working on translational fluorescence imaging and related projects. Pogue was leading an international committee tasked with recommending standards for fluorescence surgical guidance. While the commercial opportunity was not well defined, there was a clear need for better tools for characterizing fluorescence imaging systems. Our initial SBIR proposal, aimed at demonstrating feasibility, became the foundation of QUEL Imaging and the growing range of products and services we now offer across biomedical optics applications.

QUEL has a double meaning. In French, *quel* means “what” or “which,” reflecting our focus on helping customers better understand and quantify what they are imaging. It also stands for “QUantitative Enlightenment of Luminescence,” capturing our original focus on quantitative measurements of fluorescence and other luminescent signals. Since then, our work has expanded to optical imaging more broadly. Today, we are based in Vermont, with Ethan serving as CEO and Alberto as CTO. Our multidisciplinary team develops optical tissue phantoms and reference targets, along with characterization, custom design, and related services for biomedical optics.

## 2 Technology Description

Optical phantoms are materials designed to mimic the way biological tissues interact with light, particularly through absorption and scattering. Reference targets provide controlled optical properties or signals that enable quantitative characterization of imaging system performance. An overview of our product and services portfolio is shown in Fig. 1 below.

**Fig. 1:**
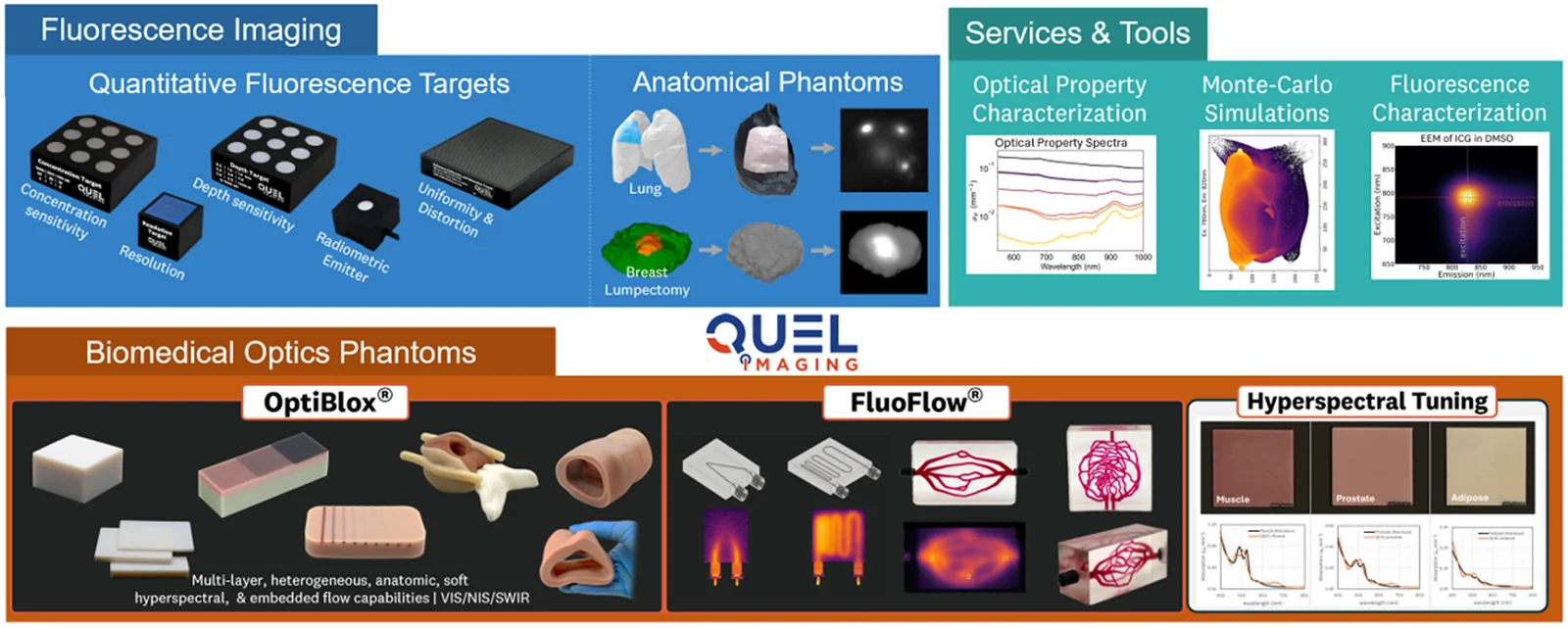
QUEL Imaging’s portfolio of optical phantoms and modeling tools provides traceable, quantitative tools for industrial and academic customers.

We manufacture both standard and custom phantoms, ranging from simple geometries to complex structures that mimic human anatomy, such as the colon and prostate. The majority of our phantoms are customized to meet application-specific needs.

Characterization is central to our approach, ensuring that customers can rely on well-defined and quantitatively measured phantom properties. We characterize absorption and reduced scattering, as well as fluorescence properties where applicable. Our standard characterization can span wavelengths from 400 to 2000 nanometers, enabling multispectral and hyperspectral applications. We also evaluate stability over time so customers can understand how closely the phantom they use continues to match our original characterization.

## 3 Clinical Need and Market

Biomedical optical imaging systems require testing throughout their development and commercialization, but researchers and companies have traditionally made many of their own phantoms. This can require specialized materials, laboratory space, and skilled personnel, while differences in preparation can make it difficult to reproduce measurements between laboratories, development sites, or over time.

Our primary customers are therefore not patients or hospitals, but companies developing and commercializing imaging systems and contrast agents. Academic groups account for about 14 percent of our sales. The majority comes from companies ranging from startups to established businesses that are advancing biomedical optics technologies toward clinical use.

Our phantoms and reference targets support these technologies throughout their lifecycle, from early development and performance characterization to validation, manufacturing, and quality assurance. They enable developers to assess sensitivity, imaging depth, resolution, distortion, uniformity, and other performance characteristics. Reference targets also provide a common basis for comparing system performance across laboratories, systems, manufacturing facilities, and clinical study sites.

## 4 Commercialization Stage and Product Development

We began attracting customers relatively early. Companies developing fluorescence imaging systems approached us about purchasing prototypes, while our published research on phantom manufacturing generated additional interest from customers looking to purchase the phantoms we had developed.

Since then, we have expanded from standard fluorescence reference targets into custom phantoms, characterization, consulting, and regulatory-support services. We have also developed an internal quality system covering components, vendors, manufacturing, and characterization, and can provide certificates of conformance or verification with our targets.

More recently, we acquired phantom-related assets, historical characterization data, and measurement equipment from the National Optics and Photonics Institute (INO) in Quebec, after it stopped producing phantoms. The technology complements our existing capabilities while preserving measurement equipment and data associated with phantoms that have been used by biomedical optics researchers for years.

## 5 Competitive Landscape

The specialized nature of optical phantom manufacturing means that our competitive landscape is relatively small. INO historically supplied phantoms for optical-system characterization, while BioPixS in Ireland also produces phantoms. When INO exited the business, we acquired some of its phantom-related assets and now receive referrals from INO when customers seek these products.

Our approach combines standardized products that customers can purchase quickly with advanced, custom manufacturing capabilities at the forefront of optical phantom development. These include heterogeneous, hyperspectral phantoms, support for multiple fluorescence regimes, and designs tailored to specialized applications. We also provide characterization and consulting services, allowing us to support customers beyond the physical phantom itself.

## 6 Funding History

Our early funding began with a Phase I SBIR award from the National Institute of Biomedical Imaging and Bioengineering (NIBIB), which helped kickstart the commercialization of our fluorescence reference targets. We subsequently received a second NIBIB SBIR grant to further advance our technologies and capabilities.

We have also undertaken federally supported R&D projects. An NCI SBIR contract supported the development of more biomimetic phantoms, while an ARPA-H project has involved work with Dartmouth College and Johns Hopkins University on testing a dual-channel fluorescence imaging system

Government grants and contracts have been instrumental in developing our technologies, manufacturing techniques, and infrastructure. In parallel, we have focused on bringing these advances to market, with commercial sales becoming an increasingly important source of revenue and enabling us to support a growing number of industry and academic customers. This combination of R&D investment and commercialization has allowed us to grow organically while accelerating the translation of biomedical optical technologies.

## 7 Regulatory Pathway

Our products do not themselves have a specific FDA regulatory pathway. Instead, they can help medical-device and contrast-agent developers characterize products that are moving through regulatory review.

For example, developers can use our phantoms to compare the performance of a new fluorescence imaging system with a predicate device and generate characterization data for an FDA submission. We also work with both imaging-device companies and pharmaceutical companies developing fluorescent contrast agents, giving us insight into the characterization challenges encountered by both sides.

Standardization remains an open problem. Imaging systems have widely different configurations, and even measurements of basic optical properties such as absorption and reduced scattering do not yet have universally agreed standards. Through a Cooperative Research and Development Agreement with NIST, we are working toward optical-property measurements traceable to SI units. We also contribute to broader efforts surrounding fluorescence-imaging characterization, including AAPM Task Group 311.

## 8 Lessons Learned

One of our most important lessons has been to understand the problem before becoming too attached to a technical solution. Through an NIH I-Corps program, we interviewed roughly 100 stakeholders. Those conversations changed how we viewed our business. Initially, we thought primarily about helping customers characterize imaging systems. We came to recognize that our greatest value lies in accelerating the translation of biomedical optical technologies by supporting their entire development lifecycle, from early R&D and validation to clinical translation and surgical training.

That experience reinforced the importance of talking with potential customers, listening carefully, and remaining willing to adapt. Scientists and engineers can easily become enthusiastic about an elegant technology before establishing whether it solves a problem that customers actually have.

We have also learned that translating technology requires a strong team and skills well beyond science and engineering. Building QUEL has required us to learn about manufacturing, quality management, marketing, sales, hiring, and company culture. For us, commercialization has become an extension of scientific translation: a way to turn research into practical tools that help other biomedical optics technologies move toward the clinic.

__

## Supplemental Information

The interview on which this report is based is available in podcast form at Biophotonics Discovery: The Podcast, available wherever you get your podcasts.

